# Risk Factors for Anti-Glaucoma Surgery in Posner-Schlossman Syndrome Patients: an 8-Year Retrospective Study

**DOI:** 10.7150/ijms.131284

**Published:** 2026-03-30

**Authors:** Jiaxing Xie, Ruofan Li, Qianqian Ji, Haoyuan Yang, Jingfei Bin, Chi Zhang, Jingyi Ren, Ying Hong

**Affiliations:** 1 Department of Ophthalmology, Peking University Third Hospital, 49 North Garden Road, Haidian District, Beijing 100191, China; 2 Beijing Key Laboratory of Restoration of Damaged Ocular Nerve, Peking University Third Hospital, 49 North Garden Road, Haidian District, Beijing 100191, China; 3 Eye Center, The Second Affiliated Hospital, School of Medicine, Zhejiang University, Hangzhou, 310009, China.; Jiaxing Xie, Ruofan Li and Qianqian Ji contributed equally to this work and shared the first authorship.

**Keywords:** Posner-Schlossman syndrome, frequency of attacks, anti-glaucoma surgery, risk factors

## Abstract

This study identifies older age, higher attack frequency and RNFL defect as risks for anti-glaucoma surgery in 106 Chinese Posner-Schlossman Syndrome (PSS) patients via an 8-year retrospective study.

**Purpose:**

To investigate the risk factors for anti-glaucoma surgery in Chinese patients with PSS.

**Patients and Methods:**

Retrospective study**.** All PSS patients visiting Peking University Third Hospital Department of Ophthalmology between June 2016 and May 2023 with a minimum follow-up of 12 months were enrolled. Patients were divided into low-frequency group and high-frequency group (whether average attacks were less than 2 times/year or not). General information, ocular manifestation and medical treatment were compared between the two groups. Risk factors for anti-glaucoma surgery were analyzed.

**Results:**

Among 106 patients, 62 cases were in the low-frequency group and 44 were in the high-frequency group. Patients in high-frequency group had a longer duration of attacks, more iris atrophy, and systemic administration of ganciclovir (p = 0.044, 0.044, 0.012, respectively). They also had a higher tendency to develop secondary glaucoma and more probability of undergoing anti-glaucoma surgery (p = 0.014, 0.017 respectively). 18 cases (17%) received anti-glaucoma surgery while binary logistic regression showed that the risk factors for receiving anti-glaucoma surgery were elder age of onset (odds ratio (OR) = 1.080; 95% confidence interval (CI): 1.012-1.153; p = 0.021), higher frequency of attacks (OR = 6.186; 95% CI: 1.276, 29.976; p = 0.024), and RNFL defect (OR = 4.804; 95% CI: 1.040, 22.184; p = 0.044).

**Conclusion:**

Risk factors for anti-glaucoma surgery in patients with PSS were elder age of onset, higher frequency of attacks and RNFL defect.

## Introduction

Posner-Schlossman syndrome (PSS), also known as glaucomatocyclitic crisis, was first described by Posner and Schlossman in 1948^1^. It is characterized by unilateral, recurrent acute elevated intraocular pressure (IOP) and mild non-granulomatous anterior uveitis. At present, it is supposed that PSS is caused by various infectious microorganisms and their specific components, such as cytomegalovirus (CMV), acting as foreign antigens in individuals with specific genetic backgrounds and activating immune cells that are mainly composed of T cells 2-4. Currently, the treatment of PSS mainly relies on steroids and anti-viral therapy (usually ganciclovir) 5, 6. However, there are still some patients who need surgical intervention to control the IOP. It was reported that 9 out of 52 patients (17.3%) required anti-glaucoma surgery, and revealed that higher peak IOP and thinner retinal nerve fiber layer (RNFL) thickness are indicators for the option for surgical treatment 7-9. Therefore, we divided 106 Chinese PSS patients enrolled from 2016-2023 into low-frequency group and high-frequency group by whether average attacks were less than 2 times/year or not. The study aimed to compare clinical characteristics between the two groups and to investigate the risk factors associated with the requirement for anti-glaucoma surgery in patients with PSS.

## Materials and Methods

### Participants

All patients visiting Peking University Third Hospital Department of Ophthalmology between June 2016 and May 2023 with a minimum follow-up of 12 months were enrolled. Gender, age of onset, frequency and duration of attacks, best-corrected visual acuity (BCVA), and IOP were recorded. Slit-lamp biomicroscope, fundus photography, visual field, optical coherence tomography and specular microscopy were recorded. Medical treatment including eyedrops, systemic medications and surgery treatment were recorded. The study was approved by the Institutional Review Board of Peking University Third Hospital (Approved No. IRB 201416605) and informed consent was obtained from all patients.

### Inclusion and exclusion criteria

Inclusion criteria were: (a) recurrent episodes of mild iritis associated with an elevated IOP (> 21 mm Hg), with or without diffuse corneal epithelial edema of the cornea, and a few keratic precipitates (KPs); (b) a normal IOP between attacks, and normal and open angles 10; (c) a unilateral attack; and (d) immunocompetency of the patient 11. None of the patients presented with peripheral iris anterior synechiae and posterior synechiae. We excluded patients with any known causes that may lead to inflammation, including systemic, genetic, and infectious causes. Patients were divided into high-frequency group (average attacks ≥ 2 times/year) and low-frequency group (average attacks < 2 times/year) during the follow-up.

### Ocular examination

Cornea and iris were observed using a slit-lamp biomicroscope. The cup disk ratio (CDR) was calculated from fundus photography by two senior glaucoma specialists. The mean deviation (MD) and pattern standard deviation (PSD) were recorded using a Humphrey automatic field analyzer (HRT II RCM Heidelberg Engineering Inc., Heidelberg, Germany). Spectral domain optical coherence tomography (HRT II RCM Heidelberg Engineering Inc., Heidelberg, Germany) was adopted to evaluate the thickness of the superior, temporal, inferior, and nasal peripapillary retinal nerve fiber layer (RNFL) of the 3.5 mm diameter subfield and the central papillary RNFL. The corneal endothelial cell (CEC) density was measured by specular microscopy (Hightech American Industrial Laboratories, Inc., HAI Laboratories, United States).

### Treatment strategy

Anti-glaucoma medications included β-blockers, α_2_-agonists, carbonic anhydrase inhibitors or prostaglandins eyedrops for 2-6 weeks (0.5% BETAGAN BID, 0.2% ALPHAGAN BID, 1.0% AZOPT BID, 0.03% LUMIGAN QN). Oral methazolamide (0.25 mg BID) and intravenous mannitol (20% 250 ml QD) may be prescribed at attack episodes. We prescribed 1% prednisolone or 0.1% fluorometholone 4 times a day and taper for anterior inflammation control, and 0.15% ganciclovir gel 4 times a day or additional oral ganciclovir (1g TID) was used for anti-viral therapy. All operations were performed during PSS attack episode. Other ocular surgery during the follow-up was also recorded.

### Statistical analysis

All data were analyzed using SPSS 28.0 (IBM SPSS Inc., Chicago, IL, United States). Variables with normal distributions are presented as mean ± standard deviation (SD) and were compared using an independent t-test between high-frequency group and low-frequency group. Variables with skewed distributions are presented as quartiles and were compared using the Mann-Whitney U-test. A binary logistic regression analysis was constructed to identify variables associated with anti-glaucoma surgery. *P* < 0.05 was considered statistically significant.

## Results

### Patient general demographics

A total of 106 patients including 49 males (46%) and 57 females (54%) were enrolled. Their age of onset was 39.0 [27.8, 50.0] years, ranging from 15 to 68 years. The BCVA of the affected eye was 0.20 [0.10, 0.40] and ranged from 0.00 to 2.10 LogMAR, while the IOP of the affected eye was 36.9 ± 10.4 (20 to 60) mm Hg.

### Comparison between high- frequency and low-frequency attacks groups

Among the 106 patients, 44 cases (42%) experienced ≥ 2 attacks and 62 cases (58%) had < 2 attacks per year**.** The difference in the duration of attacks, iris atrophy, systemic administration of ganciclovir (*p* = 0.044, 0.044, 0.012, respectively). Patients in high-frequency group had a higher tendency to develop secondary glaucoma (43% vs 21%, *p* = 0.014), while their probability of undergoing anti-glaucoma surgery was significantly increased (27% vs. 10%, *p* = 0.017). No statistical difference was found in BCVA, IOP, CDR and MD of visual field between the two groups (Table 1).

### Risk factors for anti-glaucoma surgery

Among the 106 patients, 18 cases (17%) received anti-glaucoma surgery while 88 cases (83%) did not. The demographic data and clinical characteristics of the two groups are shown in Table 2. The age of onset, high/low frequency group and whether RNFL defect were included in the logistic model. After adjusting the factors in the logistic model, the risk of anti-glaucoma surgery was higher in patients of elder age of onset (odds ratio (OR) = 1.080; 95% confidence interval (CI): 1.012-1.153; *p* = 0.021), higher frequency of attacks (OR = 6.186; 95% CI: 1.276, 29.976; p = 0.024), and RNFL defect (OR = 4.804; 95% CI: 1.040, 22.184; p = 0.044) (Table 3).

## Discussion

Our study demonstrated that older age of onset, higher frequency of attacks and RNFL defect were risk factors for PSS patients undergoing anti-glaucoma surgery.

Regarding the general demographic characteristics, current research was similar to previous reports, including gender^11^, age^12^, frequency of attacks per year^13^, duration of attacks^14^, BCVA^9^, IOP^5^, ^9^, ^12^, KP^2^, ^14^, ^15^, iris atrophy^16^, CDR^5^, visual field^5^, ^9^, RNFLD^5^, ^7^, CEC count^5^, ^7^, ^9^, and the incidence of developing secondary glaucoma^17^ and receiving anti-glaucoma surgery^7^. PSS affects a population with an average age between 32.6 and 58.9 years by previous study 12. Murata *et al*. reported that the BCVA of the affected eye was worse than that of the non-affected eye 9. It was also reported that females were slightly more common (56.6%) in PSS patients, while the frequency and duration of attacks were 1.46 ± 1.15 times per year and 5.13 ± 3.66 weeks, respectively 11, 13, 14. The above results were all similar to the findings in our study which were 39.0 (27.8, 50.0) years, BCVA 0.20 (0.10, 0.40) LogMAR, 36.9 ± 10.4 mm Hg, 53.8% female, 1.66 attacks per year, and 28.0 (18.5, 36.0) days duration, respectively. Unlike the initially assumed benign prognosis of PSS, the occurrence of secondary glaucoma in the affected eye has been observed in recent research. Li *et al*. showed that 7 out of 16 patients (43.8%) with PSS developed secondary open-angle glaucoma 18. Cao *et al*. found 59 cases (31.0%) of glaucomatous optic nerve damage among 190 PSS patients 17. In our study, the incidence of secondary glaucoma was 30.2%, similar with previous articles. Lenglinger *et al*. reported that 9 out of 52 patients (17.3%) required anti-glaucoma surgery 7, almost the same as the ratio (17%) in our study.

We observed a significant increase in the duration of attacks in high-frequency group. It was reported that the abnormal excessive activation of the complement system may result in relapse and a longer duration of PSS, which explained the significant correlation between the frequency and duration of attacks found in our study 2. Moreover, patients in high-frequency group had a higher probability of developing iris atrophy, which was predicted to be caused by persistent ocular hypertension rather than by tissue damage due to viruses 19. We also found that high-frequency group had a higher tendency to receive anti-glaucoma surgery. Furthermore, we analyze the risk factors for anti-glaucoma surgery in PSS patients.

We found elder age of onset, higher frequency of attacks and RNFL defect were risk factors for receiving anti-glaucoma surgery. Pathanapitoon *et al*. reported that an increase in the age of onset could lead to a prolonged duration of attacks and was significantly associated with a risk of secondary glaucoma, which might result from the impairment of aqueous humor outflow in elder eyes compared with younger eyes, leading to greater likelihood for increased IOP 20. There was another study demonstrated that an increase in the age of onset could also increase the risk of progression, leading to the need for long-term anti-glaucoma, anti-inflammatory, and anti-viral medication and an increased occurrence of secondary glaucoma and corneal endothelial damage 21. Tau is a cytosolic protein predominantly expressed in neurons and regulates microtubule stability within the axon. It was reported that the plasma level of Tau in PSS patients significantly increased, which would accumulate with age and lead to neurodegeneration 22.

We found that the frequency of attacks was a risk factor for anti-glaucoma surgery. In the process of recurrent inflammation of the anterior segment, the patients experienced irreversible damage to the optic nerve due to the cumulative effect of a high IOP 17. Tan *et al*. found that recurrent, relatively short-duration IOP spikes (35 mm Hg) were associated with peri-optic nerve head tissue hyper compliance and reduced retinal functional response to visual stimulation in rats 23. Another study evaluated the effect of intermittent elevations in IOP on the morphology of the rat optic nerve and found that consistent intermittent IOP elevations would produce early histologic glaucomatous damage with modest degeneration of retinal ganglion cells and their axons 24. The damage or death of ganglion cells, the thinning of RNFL, and the visual field defects suggested the development of secondary glaucoma, while the uncontrolled IOP ultimately led to the requirement for anti-glaucoma surgery 25.

Our study identified that RNFL defects are a significant predictor of surgical intervention in patients with PSS, which directly reflecting the structural integrity of the optic nerve. Although PSS is characterized by transient, often unilateral IOP elevations and traditionally considered a “benign” glaucoma subtype, recurrent IOP fluctuations may impose chronic mechanical and ischemic stress on the optic nerve head, leading to progressive RNFL degeneration 5, 26. As the RNFL represents the axonal tracts of retinal ganglion cells, its structural alteration provides a sensitive marker of glaucomatous damage 7, 27. Consistent with previous OCT-based studies, RNFL thinning has been strongly associated with optic nerve injury and progression of glaucoma 28, 29. Thus, surgical intervention becomes imperative to establish durable aqueous humor drainage or reduce production, with the goal of lowering IOP to a personalized "target range" that stabilizes optic nerve integrity and preserves visual fields. RNFL defect serve as an objective, reproducible, and clinically criterion for transitioning from conservative treatment to surgical in PSS patients. Instead of patients' subjective symptoms or uncontrolled IOP, both of which are insufficient to fully characterize optic nerve damage, RNFL assessment emerges as a pivotal marker. It enables timely surgical decision-making and contributes to the preservation of visual function in patients with PSS.

There were some limitations of our study. First, this was a retrospective study. Second, the sample size of our study was limited. Moreover, the virological test for the aqueous humor was not involved in current study, considering the invasive risks and relatively low positive rate of polymerase chain reaction, metagenomic deep sequencing, and ELISA in the detection of pathogens in the aqueous humor (9.1%, 13.6%, and 63.6%, respectively) 30. Large-sample randomized controlled trials are needed in the future.

## Conclusion

Risk Factors for anti-glaucoma surgery in patients with PSS were elder age of onset, higher frequency of attacks and RNFL defect.

## Figures and Tables

**Table 1:** Demographics of patients in high frequency and low frequency group

Demographics data	High-Frequency (n = 44)	Low-Frequency (n = 62)	*P*	
Gender (M/F)	23/21	26/36	0.293*	
Age of onset, years, mean ± SD	40.7 ± 12.9	38.8 ± 13.1	0.466†	
Affected eye (R/L)	18/26	34/28	0.157*	
Duration of attacks, days, Q50[Q25, Q75]	30.5 [24.0, 39.8]	27.0 [14.0, 35.5]	0.044‡	
BCVA, LogMAR, Q50 [Q25, Q75]	0.20 [0.10, 0.50]	0.10 [0.10, 0.30]	0.375‡	
IOP, mm Hg, mean ± SD	38.2 ± 10.5	35.8 ± 10.2	0.299†	
Iris atrophy (w/o)	5/39	1/61	0.044*	
CDR, Q50 [Q25, Q75]	0.60 [0.35, 0.70]	0.40 [0.30, 0.65]	0.165‡	
VF				
MD, dB, Q50 [Q25, Q75]	-3.80 [-11.1, -2.24]	-3.83 [-8.60, -1.70]	0.517‡	
PSD, dB, Q50 [Q25, Q75]	2.11 [1.49, 2.63]	1.97 [1.56, 5.47]	0.863‡	
RNFLD (w/o)	22/22	26/36	0.266*	
CEC density, cells/mm2, mean ± SD	2277 ± 780	2314 ± 683	0.874†	
Medication				
anti-glaucoma eyedrops, Q50 [Q25, Q75]	2.0 [1.0, 3.0]	2.0 [1.0, 2.0]	0.137‡	
Systemic anti-glaucoma medication, Q50 [Q25, Q75]	0.5 [0.0, 1.0]	0.0 [0.0, 1.0]	0.205‡	
ganciclovir gel(w/o)	41/3	55/7	0.438*	
ganciclovir capsule(w/o)	38/6	40/22	0.012*	
Secondary glaucoma (w/o)	19/25	13/49	0.014*	
Anti-glaucoma surgery (w/o)	12/32	6/56	0.017*	
Secondary Cataract surgery (w/o)	7/37	5/57	0.209*	

* Pearson χ^2^ tests † Independent t-test ‡ Mann-Whitney U-test Abbreviations: BCVA: best correct vision acuity; IOP: intraocular pressure; SD: standard deviation; CDR: cup disk ratio; MD: mean deviation; PSD: pattern standard deviation; RNFLD: retinal nerve fiber layer deficiency; CEC: corneal endothelial cell; w/o: with/without

**Table 2:** Comparisons of patients whether received anti-glaucoma surgery or not

Demographics data	No surgery (n = 88)	Surgery (n = 18)	*P*
Gender (M/F)	39/49	10/8	0.384^*^
Age of onset, years, mean ± SD	38.2 ± 12.5	46.4 ± 13.9	0.015^†^
Affected eye (R/L)	44/44	8/10	0.667^*^
Frequency of attacks, times/year, Q_50_ [Q_25_, Q_75_]	1.60 [0.69, 2.46]	2.80 [1.48, 3.62]	0.003^‡^
Duration of attacks, days, Q_50_ [Q_25_, Q_75_]	28.0 [18.0, 36.0]	28.0 [24.0, 35.0]	0.714^‡^
BCVA LogMAR, Q_50_ [Q_25_, Q_75_]	0.10 [0.10, 0.30]	0.40 [0.10, 0.90]	0.012^‡^
IOP, mm Hg, mean ± SD	35.9 ± 9.8	41.4 ± 11.7	0.056^†^
Iris atrophy (w/o)	2/79	3/13	0.007^*^
CDR, Q_50_ [Q_25_, Q_75_]	0.40 [0.30, 0.60]	0.70 [0.50, 0.85]	< 0.001^‡^
Visual field			
MD, dB, Q_50_ [Q_25_, Q_75_]	-3.10 [-6.48, -1.68]	-15.40 [-27.30, -5.90]	< 0.001^‡^
PSD, dB, Q_50_ [Q_25_, Q_75_]	1.97 [1.48, 2.35]	4.44 [1.94, 11.09]	0.081^‡^
RNFLD (w/o)	25/44	14/3	< 0.001^*^
CEC density, cells/mm^2^, mean ± SD	2425 ± 677	1912± 748	0.050^†^
Medication			
anti-glaucoma eyedrops Q_50_ [Q_25_, Q_75_]	2.0 [1.0, 2.0]	2.5 [1.8, 3.0]	0.013^‡^
Systemic anti-glaucoma medication Q_50_ [Q_25_, Q_75_]	0.0 [0.0, 1.0]	1.0 [0.0, 1.2]	0.022^‡^
Steroids Q_50_ [Q_25_, Q_75_]	2.0 [2.0, 2.0]	2.0 [2.0, 2.0]	0.303^‡^
ganciclovir gel(w/o)	80/8	16/2	0.789^*^
ganciclovir capsule(w/o)	63/25	15/3	0.303^*^
Secondary glaucoma (w/o)	14/74	18/0	< 0.001^*^
Secondary Cataract surgery (w/o)	4/84	8/10	< 0.001^*^

^*^ Pearson χ^2^ tests. ^†^ Independent t-test. ^‡^ Mann-Whitney U-test. Abbreviations: BCVA: best correct vision acuity; IOP: intraocular pressure; SD: standard deviation; CDR: cup disk ratio; MD: mean deviation; PSD: pattern standard deviation; RNFLD: retinal nerve fiber layer deficiency; CEC: corneal endothelial cell; w/o: with/without

**Table 3:** Binary logistic regression analysis of receiving anti-glaucoma surgery

Parameters	OR (95% CI)	*P*
Age of onset	1.080 (1.012, 1.153)	0.021
Attack group		
Low Frequency	1	-
High Frequency	6.186 (1.276, 29.976)	0.024
RNFL defect		
With	4.804 (1.040, 22.184)	0.044
Without	1	-

Abbreviations: OR: odds ratio; CI: confidence interval.

## Data Availability

The data that support the findings of this study are available from the corresponding author, [YH], upon reasonable request.

## References

[1] Posner A, Schlossman A (1948). Syndrome of unilateral recurrent attacks of glaucoma with cyclitic symptoms. Arch Ophthal.

[2] Yang MM, Sun HY, Meng T, Qiu SH, Zeng QQ, Ng TK (2021). CFH I62V as a Putative Genetic Marker for Posner-Schlossman Syndrome. Front Immunol.

[3] Igarashi N, Honjo M, Yamagishi R, Kurano M, Yatomi Y, Igarashi K (2020). Involvement of autotaxin in the pathophysiology of elevated intraocular pressure in Posner-Schlossman syndrome. Sci Rep.

[4] Zhai R, Wang Z, Sheng Q, Fan X, Kong X, Sun X (2022). Polymorphisms of the cytomegalovirus glycoprotein B genotype in patients with Posner-Schlossman syndrome. Br J Ophthalmol.

[5] Hülse P, Reitemeyer E, Rübsam A, Pleyer U, Maier AB (2023). Cytomegalovirus-positive Posner-Schlossman syndrome: to compare differences in retinal vessel area density between the affected and non-affected eye using optical coherence tomography angiography. Graefes Arch Clin Exp Ophthalmol.

[6] Ying Y, Sun Y, Sheng Q, Zhai R, Fan X, Kong X (2023). Steroid-Dependency in Posner-Schlossman Syndrome: A Suggested Topical 2% Ganciclovir and Gradual Decrement of Topical Steroid Combination Therapy from Shanghai PSS Study. Ocul Immunol Inflamm.

[7] Lenglinger M, Schick T, Pohlmann D, Pleyer U (2022). Cytomegalovirus-Positive Posner-Schlossman Syndrome: Impact on Corneal Endothelial Cell Loss and Retinal Nerve Fiber Layer Thinning. American journal of ophthalmology.

[8] Shimizu A, Maruyama K, Yokoyama Y, Tsuda S, Ryu M, Nakazawa T (2014). Characteristics of uveitic glaucoma and evaluation of its surgical treatment. Clinical ophthalmology (Auckland, NZ).

[9] Murata K, Ishida K, Ozawa K, Sawada A, Mochizuki K, Yamamoto T (2019). The characteristics of Posner-Schlossman syndrome: A comparison in the surgical outcome between cytomegalovirus-positive and cytomegalovirus-negative patients. Medicine (Baltimore).

[10] Chee SP, Bacsal K, Jap A, Se-Thoe SY, Cheng CL, Tan BH (2008). Clinical features of cytomegalovirus anterior uveitis in immunocompetent patients. Am J Ophthalmol.

[11] Li J, Ang M, Cheung CM, Vania M, Chan AS, Waduthantri S (2012). Aqueous cytokine changes associated with Posner-Schlossman syndrome with and without human cytomegalovirus. PloS one.

[12] Megaw R, Agarwal PK (2017). Posner-Schlossman syndrome. Survey of ophthalmology.

[13] Antoun J, Willermain F, Makhoul D, Motulsky E, Caspers L, Relvas LJ (2017). Topical Ganciclovir in Cytomegalovirus Anterior Uveitis. J Ocul Pharmacol Ther.

[14] Sheng Q, Zhai R, Fan X, Kong X The Analysis of Dynamic Changes and Prognosis of Posner-Schlossman Syndrome with Cytomegalovirus Infection and Antiviral Therapy. J Ophthalmol. 2021: 6687929.

[15] Sheng Q, Sun Y, Zhai R, Fan X, Ying Y, Kong X (2023). 2% Ganciclovir Controlled Posner-Schlossman Syndrome Relapse and Reduced the Chance of Corticosteroid Dependence: A Large Cohort in East China. Ocul Immunol Inflamm.

[16] Warjri GB, Das AV, Senthil S (2024). Clinical profile, demographic distribution, and management of Posner-Schlossman syndrome: An electronic medical record-driven data analytics from an eye care network in India. Indian J Ophthalmol.

[17] Cao D, Hu J, Sun Z, Lei Q, Zhang W, Zhang Y (2023). Preliminary research of the clinical pathways of glaucomatous optic nerve damage in cases with glaucomatocyclitic crisis: A retrospective study. Medicine (Baltimore).

[18] Li J, Ji Y, Yang W, Yao Y, Wang S, Zhang Z (2022). Analysis of risk factors associated with secondary open-angle glaucoma in Posner-Schlossman syndrome: A retrospective case-control study. Front Med (Lausanne).

[19] Sakai JI, Usui Y, Suzuki J, Kezuka T, Goto H (2019). Clinical features of anterior uveitis caused by three different herpes viruses. Int Ophthalmol.

[20] Pathanapitoon K, Smitharuck S, Kunavisarut P, Rothova A (2017). Prevalence and Visual Outcome of Glaucoma with Uveitis in a Thai Population. J Glaucoma.

[21] Joseph DM, Lim LL, Samalia PD, Wells JM, McCluskey PJ, Paul E (2023). Long term outcome and prognostic indicators in Posner Schlossman syndrome. Clin Exp Ophthalmol.

[22] Gan YJ, Fang AW, Liu C, Liu BJ, Yang FM, Guan JT (2019). Elevated Plasma Levels of Drebrin in Glaucoma Patients with Neurodegeneration. Front Neurosci.

[23] Tan B, Gurdita A, Choh V, Joos KM, Prasad R, Bizheva K (2018). Morphological and functional changes in the rat retina associated with 2 months of intermittent moderate intraocular pressure elevation. Sci Rep.

[24] Joos KM, Li C, Sappington RM (2010). Morphometric changes in the rat optic nerve following short-term intermittent elevations in intraocular pressure. Invest Ophthalmol Vis Sci.

[25] Artini W, Bani AP (2019). The effectiveness of trabeculectomy with mitomycin C and releasable suture in posner-schlossman syndrome with secondary glaucoma: A case series. Nigerian journal of clinical practice.

[26] Nishida T, Moghimi S, Chang AC, Walker E, Liebmann JM, Fazio MA (2022). Association of Intraocular Pressure with Retinal Nerve Fiber Layer Thinning in Patients with Glaucoma. JAMA ophthalmology.

[27] Koornwinder A, Zhang Y, Ravindranath R, Chang RT, Bernstein IA, Wang SY (2025). Multimodal Artificial Intelligence Models Predicting Glaucoma Progression Using Electronic Health Records and Retinal Nerve Fiber Layer Scans. Translational vision science & technology.

[28] Shin JW, Sung KR, Song MK (2020). Ganglion Cell-Inner Plexiform Layer and Retinal Nerve Fiber Layer Changes in Glaucoma Suspects Enable Prediction of Glaucoma Development. American journal of ophthalmology.

[29] Aydin A, Wollstein G, Price LL, Fujimoto JG, Schuman JS (2003). Optical coherence tomography assessment of retinal nerve fiber layer thickness changes after glaucoma surgery. Ophthalmology.

[30] Wang L, Wang Z, Ma J, Li Q, Chen X, Chen Y (2022). Comparison of Intraocular Antibody Measurement, Quantitative Pathogen PCR, and Metagenomic Deep Sequencing of Aqueous Humor in Secondary Glaucoma Associated with Anterior Segment Uveitis. Ocul Immunol Inflamm.

